# BNTA alleviates inflammatory osteolysis by the SOD mediated anti-oxidation and anti-inflammation effect on inhibiting osteoclastogenesis

**DOI:** 10.3389/fphar.2022.939929

**Published:** 2022-09-29

**Authors:** Huidong Wang, Xiankun Cao, Jiadong Guo, Xiao Yang, Xiaojiang Sun, Zhiyi Fu, An Qin, Yujie Wu, Jie Zhao

**Affiliations:** Shanghai Key Laboratory of Orthopedic Implants, Department of Orthopaedics Surgery, Shanghai Ninth People’s Hospital, Shanghai Jiao Tong University School of Medicine, Shanghai, China

**Keywords:** BNTA, MAPK, SOD, osteolysis, inflammatory osteolysis, bone loss

## Abstract

Abnormal activation and overproliferation of osteoclast in inflammatory bone diseases lead to osteolysis and bone mass loss. Although current pharmacological treatments have made extensive advances, limitations still exist. N-[2-bromo-4-(phenylsulfonyl)-3-thienyl]-2-chlorobenzamide (BNTA) is an artificially synthesized molecule compound that has antioxidant and anti-inflammatory properties. In this study, we presented that BNTA can suppress intracellular ROS levels through increasing ROS scavenging enzymes SOD1 and SOD2, subsequently attenuating the MARK signaling pathway and the transcription of NFATc1, leading to the inhibition of osteoclast formation and osteolytic resorption. Moreover, the results also showed an obvious restrained effect of BNTA on RANKL-stimulated proinflammatory cytokines, which indirectly mediated osteoclastogenesis. In line with the *in vitro* results, BNTA protected LPS-induced severe bone loss *in vivo* by enhancing scavenging enzymes, reducing proinflammatory cytokines, and decreasing osteoclast formation. Taken together, all of the results demonstrate that BNTA effectively represses oxidation, regulates inflammatory activity, and inhibits osteolytic bone resorption, and it may be a potential and exploitable drug to prevent inflammatory osteolytic bone diseases.

## Introduction

The homeostasis of bone metabolism is finely regulated and maintained based on the balance of osteogenesis by osteoblasts and osteolysis by osteoclasts. Inflammatory bone diseases, usually caused by some infectious bone diseases, such as purulent spondylitis, purulent osteomyelitis, and periprosthetic infection, induce abnormal activation and overproliferation of osteoclasts and eventually lead to osteolysis and bone mass loss (Kim et al., 2013). Current studies have found that the release of endogenous products of bacteria can activate immune cells, cause excessive proliferation of osteoclasts and enhance bone resorption (Mbalaviele et al., 2017; Yang et al., 2021). Over the past few decades, there have been some advances in the treatment of inflammatory osteolytic bone diseases, but some limitations remain. Estrogen is mainly used to treat osteoporosis in postmenopausal women, but it carries a risk of breast cancer (Clemons and Goss, 2001; Lobo, 2017); bisphosphonates are used to inhibit osteoclastic bone resorption, but they are increasingly reported to be associated with the occurrence of aseptic necrosis of the mandible and local fractures (Woo et al., 2006; Shane et al., 2014); parathyroid hormone increases bone formation by promoting osteoblast activity, but it cannot be taken orally and cannot be used for a long time because it risks causing bone tumors (Nikitovic et al., 2016; Al-Khan et al., 2021). Therefore, there is still a great need to develop more effective and safe anti-osteolytic drugs.

Osteoclasts, acting with a vital role in osteolytic bone diseases, are large multinucleated phagocytes that originate from mononuclear-macrophage lines. The differentiation and maturity from bone marrow monocyte/macrophage to osteoclasts are dependent on the receptor activator of nuclear factor kappa B ligand (RANKL), a direct regulatory factor that controls the dynamic differentiation processes of osteoclasts (Lagasse and Weissman, 1997; Theill et al., 2002). The macrophage colony-stimulating factor (M-CSF), a cytokine for the proliferation and survival of osteoclast precursor cells, also plays an essential role in osteoclastogenesis (Wiktor-Jedrzejczak et al., 1990; Lagasse and Weissman, 1997). The binding of RANKL with its receptor the receptor activator of nuclear factor kappa B (RANK) initiates the recruitment of TNF receptor-associated factor 6 (TRAF6), which then activates multiple downstream signaling including mitogen-activated protein kinases (MAPKs) and NF-κB pathways, leading to the activation of c-fos and nuclear factor of activated T cells 1 (NFATc1). These signaling cascades enable the expression of osteoclast-related genes which administrate osteoclast fusion, cytoskeletal reorganization, and resorptive function (Chen X. et al., 2019). Among the cytokines regulating osteoclast activation and formation, RANKL is the most important one (Lee et al., 2019). Thus, interrupting the interaction between RANK and RANKL is an efficient and feasible strategy for suppressing osteoclast differentiation and maturation (Xu et al., 2021).

Reactive oxygen species (ROS), including hydroxyl radicals or superoxide anions, play a very important role in regulating the differentiation of osteoclasts. Previous research has suggested that the target molecule ROS stimulated and facilitated osteoclast differentiation and osteolytic resorption through activating the downstream MAPKs pathway and subsequently switching on the crucial NAFTc1 signals (Lee et al., 2005; Zhan et al., 2019; Zhang et al., 2019). Under normal physiological conditions, ROS in osteoclasts activate osteoclastogenesis and facilitate bone resorption (Agidigbi and Kim, 2019). However, excessive ROS in osteoclasts, not only causes direct or indirect oxidative damage, impairing DNA and proteins but also leads to osteolysis and bone destruction (Wells et al., 2009; Kanzaki et al., 2017). The ROS physiological homeostasis depends on the balance between ROS generation and removal rate by scavenging (Maciag et al., 2013; He et al., 2021). The SODs are the first and most important antioxidant enzyme defense system, which are against ROS, especially superoxide anion radicals (Zelko et al., 2002). There have three distinct isoforms of SODs been identified in mammals at present, including SOD1 (CuZn-SOD, located in the cytoplasm), SOD2 (Mn-SOD, located in the mitochondrial), and SOD3 (EC-SOD, located in the extracellular spaces) (Zelko et al., 2002). SOD3 is the most recently discovered member of the SOD family, which exhibited high expression in selected tissues, particularly in blood vessels, lungs, kidneys, and heart (Fattman et al., 2003; Nozik-Grayck et al., 2005). It can regulate proinflammatory factor expression and affect the inflammatory response, but there is almost no report on the effect of SOD3 on osteoclastogenesis (Kemp et al., 2010; Hu et al., 2019). Unlike SOD3, SOD1 and SOD2 express practically in all cells (Wang et al., 2018). Several researches have focused on the possible role of SODs on osteolytic disease. The deficiency of SOD1 or SOD2 lead to increased osteoclastogenesis *in vitro* and decreased bone volume *in vivo*, while the upregulation of SOD1 or SOD2 reduced osteoclastogenesis by reducing ROS level (Lee et al., 2014; Kobayashi et al., 2015; Lee and Jang, 2015; Kim et al., 2017; He et al., 2021).

BNTA is a small artificial synthesized molecule compound. It was firstly reported that BNTA not only effectively activates the SOD3-mediated dismutation reaction of superoxide anions but also obviously inhibited the expression of inflammatory factors in osteoarthritis (OA) (Shi et al., 2019). Given its significant bioactivation and the pivotal role of inflammation and oxidation on osteolytic bone diseases, we hypothesized that BNTA may affect the osteoclastic formation and osteolytic resorption, similar to the effect on chondrocyte and OA, by regulating the SOD (SOD1 and SOD2)-ROS-MAPK signaling pathways axis and SOD-ROS-inflammation axis. In this study, we evaluated the therapeutic effects of BNTA on RANKL-induced osteoclastogenesis *in vitro* and lipopolysaccharides (LPS)-induced inflammatory osteolysis in mice calvaria *in vivo*, focusing on clarifying the antioxidant capacity of BNTA and elaborating its underlying mechanisms.

## Materials and methods

### Chemicals and reagents

The synthetic small molecule compound BNTA (CAS: 685119-25-9), purchased from National Compound Resource Center (Shanghai, China) and Maybridge (Cornwall, Cornwall, United Kingdom), was dissolved by Dimethyl sulfoxide (DMSO) and stored at −20°C. It was diluted in a cell culture medium so that the final concentrations of DMSO were less than 0.1%.

### Bone marrow macrophage preparation and *in vitro* osteoclast differentiation

Primary bone marrow-derived monocytes/macrophages (BMMs) were isolated from the femoral and tibial bone marrow of 4-weeks-old C57BL/6J mice. Isolated cells were then cultured in α-minimum essential medium (α-MEM) supplemented with 10% fetal bovine serum (FBS), 1% penicillin/streptomycin (Gibco, Thermo Fisher Scientific, Waltham, MA, United States), and 30 ng/ml M-CSF (R&D, Systems MN, United States) in an incubator with 5% CO_2_ and 95% air at 37°C until cells reached 90% confluence. BMMs were seeded into a 96-wells plate at a density of 8,000 cells/well, and cultured in a complete α-MEM medium with 30 ng/ml M-CSF, 50 ng/ml RANKL (R&D, Systems MN, United States), and different concentrations of BNTA (10, 20, and 40 μM). Untreated BMM cells were also included as positive controls and each concentration had three replications. The culture medium was replaced every 2 days. Mature, large, multinucleated osteoclasts were formed after incubation for a total of 5–7 days. Cells were gently washed twice, fixed with 4% paraformaldehyde (PFA) for 15 min, and then stained for tartrate-resistant acid phosphatase (TRAP). The number and size of TRAP-positive multinucleated osteoclasts with at least three nuclei were scored.

### Cytotoxicity assay

The cytotoxic and proliferated effect of BNTA on BMMs was assessed using the Cell Counting Kit-8 (CCK-8) assay (Dojindo, Kumamoto, Japan). To determine toxicity, BMMs were seeded into a 96-well plate at a density of 8 * 10^3^ cells/well and cultured with different concentrations of BNTA (0, 1, 10, 20, 40, 80, and 160 μM) for 24 h. To determine proliferation, BMMs at a density of 3*10^3^ cells/well were cultured with BNTA for 24, 48, 72, and 96 h. After being seeded into a 96-well plate for 24 h to allow them to adhere, BMMs were then treated with different concentrations of BNTA (0, 1, 10, 20, and 40 μM). The cultural medium was changed every second day. At the end of the experimental period, 10 μL CCK-8 buffer was added to each well, and the plate was incubated for an additional 2 h. The absorbances were read at a wavelength of 450 nm by a spectrophotometer on an Infinite M200 Pro multimode microplate reader (Tecan Life Sciences, Männedorf, Switzerland).

### *In vitro* osteoclast bone absorption assay

Bone resorption effects of osteoclasts were measured with a hydroxyapatite-coated plate (Corning Inc. NY, United States). BMMs were seeded onto hydroxyapatite-coated plates in triplicates with a concentration of 5×10^3^/well. Cells were cultured for 24 h to allow adherence and then induced to osteoclasts with 50 ng/ml RANKL, with or without different concentrations of BNTA (10, 20, and 40 μM). Culture medium was replaced every 2 days. After 7 days, the cells were brushed off the plates, and resorption pits were pictured with a light microscope (OLYMPUS, IX71). The area of resorption pits was measured using ImageJ software.

### RNA extraction and quantitative real-time PCR

Quantitative real-time PCR was used to measure the gene expression levels during osteoclast formation. Mouse BMMs were cultured in six-well plates at a concentration of 5×10^3^/well with complete α-MEM including 30 ng/ml M-CSF and 50 ng/ml RANKL. During the RANKL-induced osteoclastogenesis, BMMs were treated with different concentrations of BNTA (10, 20, and 40 μM). Untreated samples were used as controls. The total RNA of each sample was extracted using a TRIzol reagent (Thermo Fisher Scientific, Waltham, MA, United States) after BNTA treatment for 5 days. Complementary DNA (cDNA) was synthesized with 1 μg of RNA extracted from each sample, 4 μL of 5× PrimeScript RT Master Mix (TaKaRa Bio, Otsu, Japan) and RNAse-free distilled water (dH_2_O) in a total volume of 20 μL. The real-time qPCR was performed using the TB Green Premix Ex Taq kit (TaKaRa Bio, Otsu, Japan) on an SYBR Green-Based Real-Time Quantitative Reverse Transcription PCR System (Thermo Fisher Scientific, Waltham, MA, United States). Primers were shown in Table 1. Target gene expression levels were determined using the 2^−ΔΔCT^ method and normalized to the expression of GAPDH.

**TABLE 1:** Gene primer sequences used in qPCR.

Target genes	Accession number	Primer sequences 5’→3′
CTSK	NM_007802.4	F: TAG​CCA​CGC​TTC​CTA​TCC​GA
		R: CCT​CCG​GAG​ACA​GAG​CAA​AG
NFATc1	NM_001164112.1	F: CTT​CGA​GTT​CGA​TCA​GAG​CGG
		R: AGG​GTC​GAG​GTG​ACA​CTA​GG
ACP5	NM_001102405.1	F: CAC​TCC​CAC​CCT​GAG​ATT​TGT
		R: CAT​CGT​CTG​CAC​GGT​TCT​G
D2	NM_175406.3	F: GCA​GAG​CTG​TAC​TTC​AAT​GTG​G
		R: TAG​TCC​GTG​GTC​TGG​AGA​TG
c-Fos	NM_010234.3	F: TGT​TCC​TGG​CAA​TAG​CGT​GT
		R: TCA​GAC​CAC​CTC​GAC​AAT​GC
MMP9	NM_013599.5	F: CCC​TGG​AAC​TCA​CAC​GAC​AT
		R: TGG​TTC​ACC​TCA​TGG​TCC​AC
SOD1	NM_011434.2	F: TCT​CGT​CTT​GCT​CTC​TCT​GG
		R: CTT​GCC​TTC​TGC​TCG​AAG​TG
SOD2	NM_013671.3	F: CTG​TCC​GAT​GAT​GTC​AGC​CA
		R: AAC​CCA​TTT​GCC​GCT​ACT​GA
SOD3	NM_011435.3	F: GAG​AAG​ATA​GGC​GAC​ACG​CA
		R: GAG​AAC​CAA​GCC​GGT​GAT​CT
TNF-α	NM_001278601.1	F: GCC​TCT​TCT​CAT​TCC​TGC​TTG​TGG
		R: TGG​TTT​GTG​AGT​GTG​AGG​GTC​TG
IL-1β	NM_008361.4	F: TCG​CAG​CAG​CAC​ATC​AAC​AAG​AG
		R: AGG​TCC​ACG​GGA​AAG​ACA​CAG​G
IL-6	NM_001314054.1	F: CTG​GTC​TTC​TGG​AGT​ACC​ATA​GC
		R: GTG​ACT​CCA​GCT​TAT​CTC​TTG​GT
IL-4	NM_021283.2	F: ATG​GAT​GTG​CCA​AAC​GTC​CT
		R: AAG​CCC​GAA​AGA​GTC​TCT​GC
GAPDH	NM_008084.3	F: ACC​CAG​AAG​ACT​GTG​GAT​GG
		R: CAC​ATT​GGG​GGT​AGG​AAC​AC

### Western blot analysis

To assess the effect of BNTA on the signaling pathway, BMMs were seeded into six-well plates. They were starved with serum-free α-MEM medium with or without 40 μM BNTA for 2 h. After that, cells of each group were stimulated with 50 ng/ml RANKL for 10 min. To determine the influence of BNTA on c-fos and NFATc1 expression, BMMs were cultured in a complete α-MEM medium including 30 ng/ml M-CSF and 50 ng/ml RANKL, with or without 40 μM BNTA for 1, 3, or 5 days, or with different concentrations of BNTA (0, 10, 20, 40 μM) for 5 days. Total protein was extracted from cultured cells using the radioimmunoprecipitation assay (RIPA) lysis buffer with phosphatase and protease inhibitor cocktail (Roche, Basel, Switzerland). The proteins were separated using 4%–20% SDS-polyacrylamide gel electrophoresis (PAGE) gel (GenScript Laboratories, Piscataway, NJ, United States) electrophoresis, and were then transferred onto a polyvinylidene fluoride (PVDF) membrane (Merck Millipore, CA, United States). The membranes were blocked with 5% skim milk-TBST at RT for 1 h. After being washed 3 times for 15 min, the membranes were incubated overnight at 4°C with primary antibodies (Cell Signal Technology, Danvers, MA, United States) against the following proteins: p38 MAPK (D13E1), phospho-p38 MAPK (Thr180/Tyr182; D3F9), p44/42 MAPK (ERK1/2; 137F5), phospho-p44/42 MAPK (ERK1/2; Thr202/Tyr204; D13.14.4E), SAPK/c-Jun N-terminal kinase (JNK;), phospho-SAPK/JNK (Thr183/Tyr185; 81E11), NFAT2 (D15F1); c-Fos (9F6); β-actin (D6A8). The membranes were washed by TBST 3 times and then incubated with anti-rabbit or anti-mouse IgG secondary antibody (H + L; DyLight™800 4×PEG conjugate; Cell Signaling Technology, Danvers, MA, United States) for 1 h in dark. Finally, the membranes were washed another 3 times for 15 min, and the protein bands were visualized by the LI-COR Odyssey fluorescence imaging system (LI-COR Biosciences, Lincoln, NE, United States). The gray value of each protein band was quantified by ImageJ software.

### Intracellular ROS generation assay

The intracellular production of ROS was quantified using DCFDA cellular ROS Assay Kit (Beyotime, Shanghai, China) following the manufacturer^’^s instruction and as described previously (Lee et al., 2005). BMMs were cultured in 96-well plates at a concentration of 8×10^3^/well with complete α-MEM supplementing with 30 ng/ml M-CSF, 50 ng/ml RANKL, and different concentrations of BNTA (0, 10, 20, 40 μM) for 72 h. Cells were then washed and incubated in DCFH-DA solution (5 mM) diluted with Hank^’^s buffer for 1 h. Subsequently, the digital images of each well were taken by fluorescence microscopy (Nikon Eclipse C1, NIKON, Japan). The number of ROS-positive cells per well was analyzed with ImageJ software.

### LPS-induced calvarial osteolysis model

The animal experiments were consented to by the Animal Care and Experiment Committee of Shanghai Jiao Tong University of Medicine. Procedures of all experiments were performed in strict accordance with the guidelines for Ethical Conduct in the Care and Use of Nonhuman Animals in Research by the American Psychological Association. Twenty-four 7-weeks-old C57BL/6J male mice were randomized into four groups: 1) sham-operated control group (PBS-treated); 2) LPS control group (LPS 5 mg/kg); 3) low dose BNTA group (LPS 5 mg/kg and BNTA 0.15 mg/kg); 4) high dose BNTA group (LPS 5 mg/kg and BNTA 1.5 mg/kg). Gelatin sponges (4 mm × 4 mm×2 mm) soaked with PBS or LPS were implanted on the midline suture of the skull surfaces to induce osteolysis. Next, PBS or LPS was injected into the gelatin sponges on the surfaces of the skulls, and BNTA was administered by abdominal injections every second day for 10 days. When arrived the deadline, all groups of mice were euthanized and then their calvarial bones were separated.

### μCT scanning

A high-resolution Micro-CT instrument (Skyscan; Burker, Kontich, Belgium; resolution 10 μm; X‐ray source 46 kv/75 μA; exposure time 80 ms applied; conducted in 75% ethanol) was used for calvarias scanning. A square around the osteolytic area was defined as an interesting region for bone volume analysis. Trabecular morphometry was analyzed by measuring the bone volume fraction (BV/TV), trabecular thickness (Tb.Th), trabecular number (Tb.N), and trabecular spacing (Tb.Sp).

### Histomorphometry and immunofluorescence assay

The calvarial bones were then fixed with 4% polyformaldehyde and decalcified with 10% EDTA, buried in paraffin, and cut into 4 mm thick slices. Slices were used to make HE and TRAP staining. After taking pictures with Axio ScopeA1 light microscope (ZEISS, Germany), quantitative analysis was performed using ImageJ software. Furthermore, we detected redox-related and inflammation-related proteins using immunofluorescence assays. The tissue slices were incubated with primary antibodies overnight against the proteins as follows: SOD1 (10269-1-AP, Proteintech, United States), SOD2 (24127-1-AP, Proteintech, United States), SOD3 (DF7753, Affinity, United States), TNF-α (ab183218, Abcam, United Kingdom), IL-1β (ab216995, Abcam, United Kingdom), IL-4 (AF5142, Affinity, United States), and IL-6 (66146-1-Ig, Proteintech, United States). After being washed with PBS, slices were incubated with FITC-conjugated secondary antibodies (Proteintech, Chicago, Illinois, United States) for 1 h, and subsequently with DAPI (ab285390, Abcam, Cambridge, United Kingdom) for 15 min at room temperature. Digital fluorescence images were taken with fluorescence microscopy (Nikon Eclipse C1, NIKON, Japan). The fluorescence images were analyzed by ImageJ software. Besides, the main organs (heart, liver, spleens, lungs, and kidneys) of groups of mice were excised and histologically analyzed *via* hematoxylin and eosin (H&E) to evaluate the toxicity of BNTA *in vivo*.

### Statistical analysis

GraphPad Prism 8 (GraphPad Software, San Diego, CA, United States) was used to analyze the data. Results were presented as mean ± standard deviation (SD) with at least three replications. Statistical differences were conducted with two-tailed, unpaired Student’s *t-*test or one-way ANOVA followed by Turkey’s post hoc. *p value less than 0.05, **p value less than 0.01, ***p value less than 0.001 and ****p value less than 0.0001 were defined as statistically significant.

## Results

### Effects of BNTA on viability and proliferation of BMMs

**FIGURE 1:**
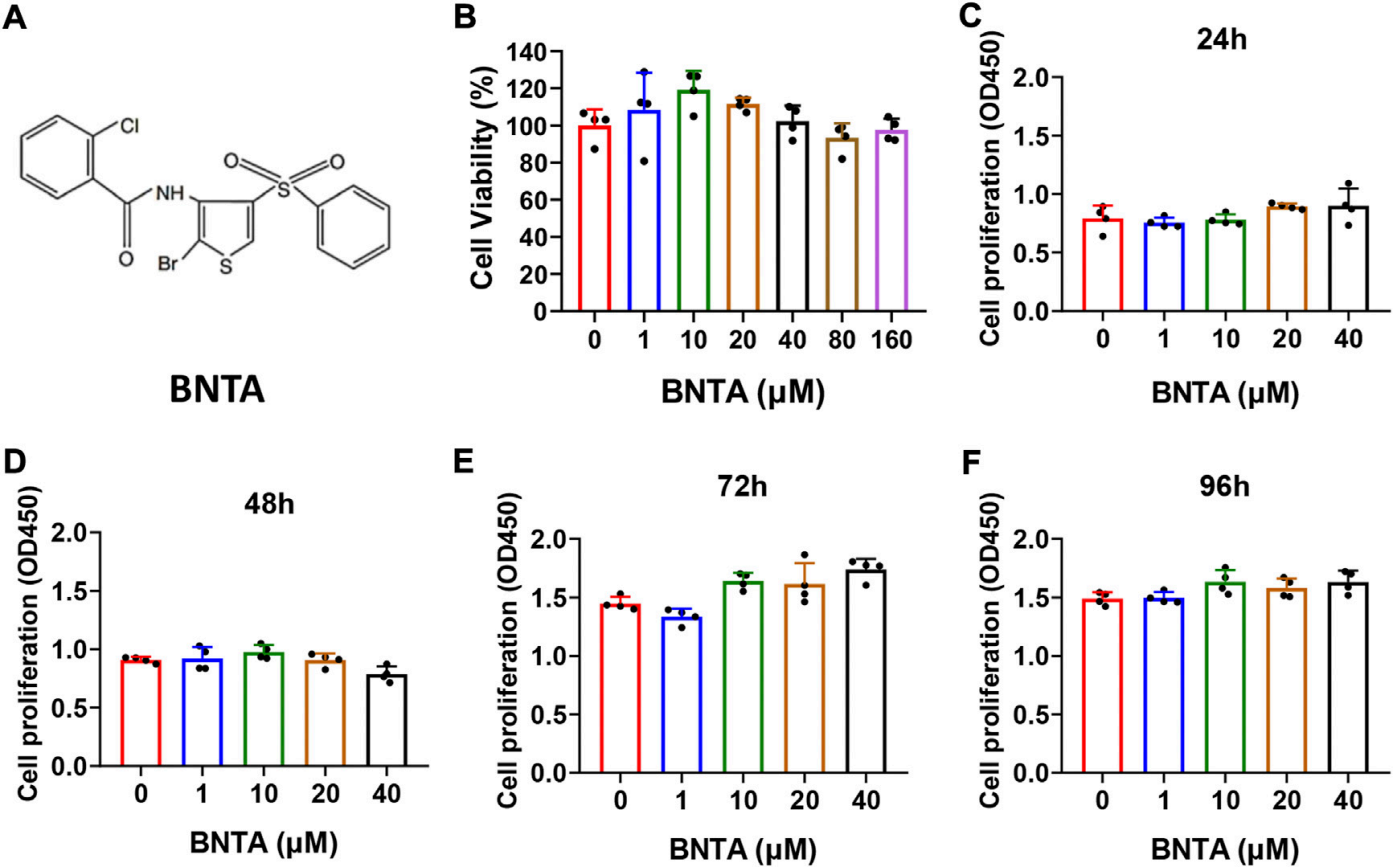
Effects of N-[2-bromo-4-(phenylsulfonyl)-3-thienyl]-2-chlorobenzamide (BNTA) on the cytotoxicity and proliferation of bone marrow-derived monocytes (BMMs). **(A)** The chemical structure of BNTA. **(B)** Viability of BMMs treated by indicated concentrations of BNTA at 24 h (*n* = 4 per group). **(C–F)** The proliferation of BMMs treated by indicated concentrations of BNTA at 24, 48, 72, and 96 h (*n* = 4 per group). All data are presented as mean ± SD.

The chemical structure of BNTA is presented in Figure 1A. Effects of BNTA on viability and proliferation of BMMs were analyzed by CCK-8 assay. Treatment of BMMs with BNTA of 0, 1, 10, 20, and 40 μM showed that BNTA had no significant effect on the viability and proliferation of BMM (Figures 1B–F). Based on the results, 40 μM of BNTA was used in the subsequent experiments.

### BNTA inhibited RANKL-induced osteoclast differentiation and impaired osteoclastic bone resorption *in vitro*

**FIGURE 2:**
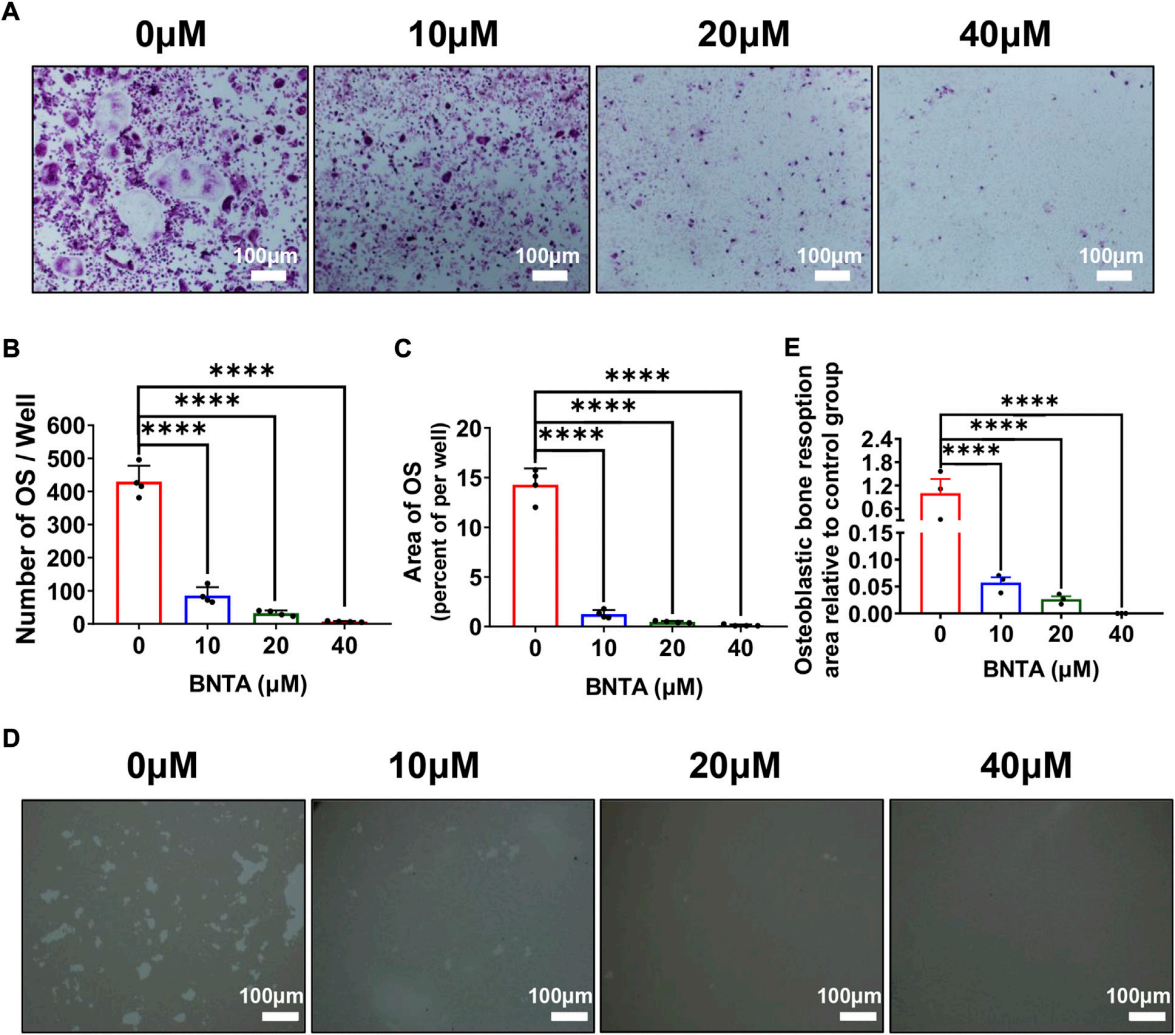
BNTA inhibited RANKL-induced osteoclastogenesis and osteolysis *in vitro*. **(A)** Representative images of BMMs stained for TRAP showed BNTA inhibited osteoclastogenesis in a dose-dependent manner. BMMs were stimulated by RANKL for 5 days with or without indicated concentrations of BNTA. **(B,C)** Quantification of TRAP-positive multinucleated cells (nuclei ≥ 3) (*n* = 4 per group). All data are presented as mean ± SD. ****p < 0.0001 compared with control group. **(D)** Representative images of hydroxyapatite resorption in each group presented BNTA inhibited osteolysis dose-dependently. BMMs were stimulated by RANKL for 5 days with or without indicated concentrations of BNTA. **(E)** Quantification of resorbed hydroxyapatite area per well. (*n* = 3 per group). All data are presented as mean ± SD. ****p < 0.0001 compared with control group.

First, the effects of BNTA on RANKL-induced osteoclastogenesis *in vitro* were analyzed. As is shown in Figure 2A that BMMs of the control group formed typical “pancake” shaped and highly TRAP-positive multinucleated osteoclasts, as was described in the previous report (Chen X. et al., 2019). The number of osteoclasts in the control group was presented to be 429.75 ± 48.20 cells per well. However, the number of osteoclasts in the 40 μM group decreased to 6.75 ± 1.71 cells per well and typical “pancake” shaped multinucleated osteoclasts almost didn’t form. The formation of osteoclasts was significantly inhibited by BNTA in a concentration-dependent mode (Figures 2B,C). Subsequently, the bone resorption function of osteoclasts was evaluated *via* Hydroxyapatite resorption experiments. Compared to the control group, the bone resorption area of groups treated with 10, 20, and 40 μM BNTA showed reductions of 94.3%, 97.3%, and 99.9%, respectively (Figures 2D,E). Thus, these data suggested that BNTA can effectively inhibit osteoclast differentiation and impair bone resorption *in vitro*.

### BNTA inhibited osteoclastogenesis and osteoclast-related genes expression

**FIGURE 3:**
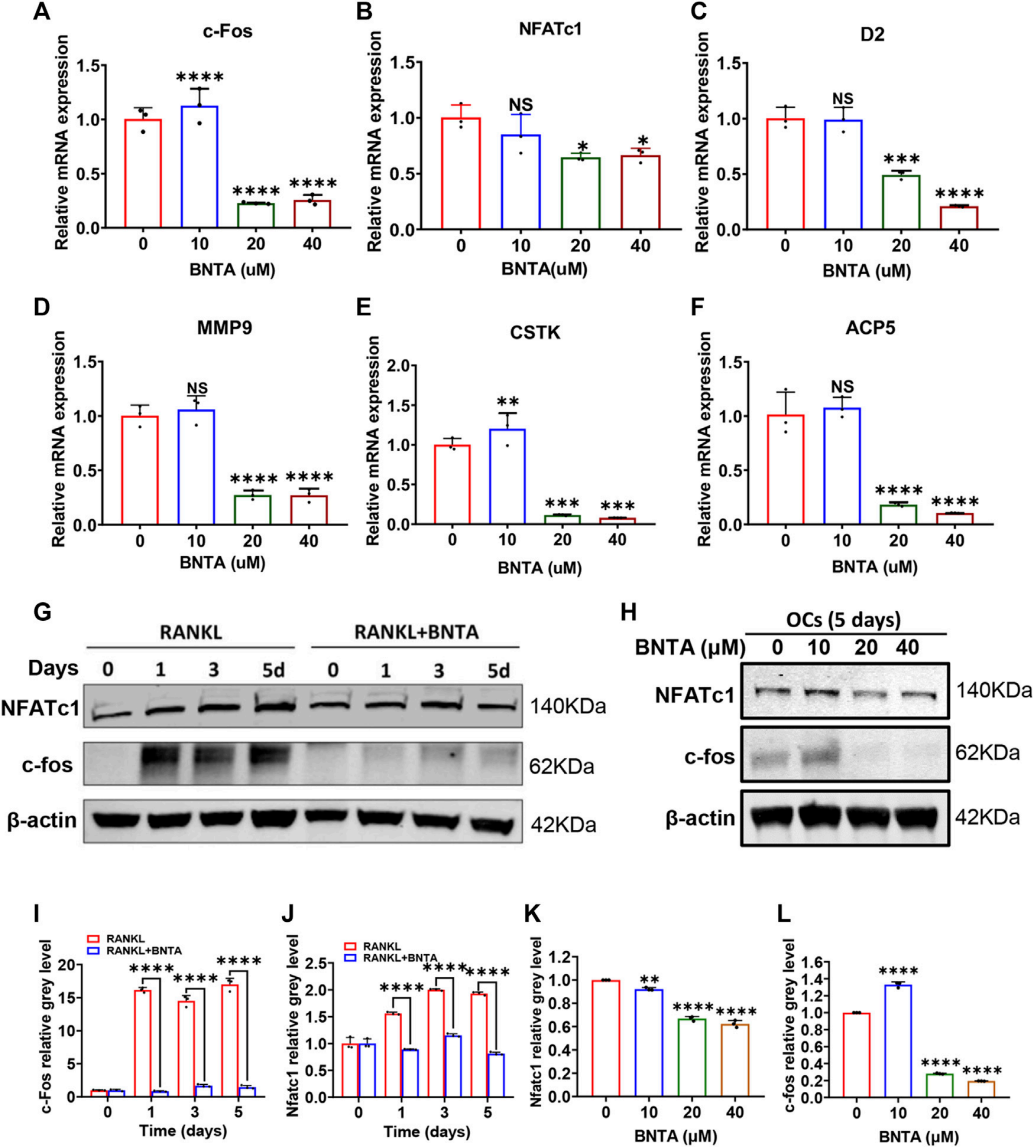
BNTA attenuated osteoclast-related gene expression and inhibited NFATc1 activation. **(A–F)** qPCR analysis of osteoclast-related gene expression of c-fos, NFATc1, D2, MM9, CSTK, and ACP5 relative to Control in BMMs stimulated by RANKL for 5 days with or without an indicated dose of BNTA (*n* = 3 per group). All data are presented as mean ± SD. *p < 0.05, **p < 0.01, ***p < 0.001, ****p < 0.0001 compared with control group. **(G)** Representative Western Blot images of the effect of BNTA on the protein expression of NFATc1 and c-fos on days 0,1, 3, and 5 with the stimulation of RANKL. **(H)** Representative Western Blot images of the effect of BNTA on the protein expression of NFATc1 and c-fos at day 5 stimulated by RANKL with or without an indicated dose of BNTA. **(I–L)** Quantitative analysis of the ratio of band intensity of NFATc1 and c-fos relative to β-actin (*n* = 3 per group). **p < 0.01, ****p < 0.0001 relative to control group.

The effects of BNTA on osteoclastogenesis and bone resorption were next determined by investigating the mRNA expression levels of osteoclastic marker genes. The quantification of osteoclastic marker genes was conducted by qPCR. The expression of genes, including c-fos, NFATc1, CTSK, D2, MMP9, and ACP5 was remarkably suppressed in 20 or 40 μM BNTA treated groups compared to the control group (Figures 3A–F). We further investigated c-fos and NFATc1 gene expressions during RANKL-induced osteoclast formation using western blot analysis. The results demonstrated that BMMs reduced c-fos and NFATc1 protein translation during the treatment of BNTA for 1, 3, or 5 days (Figures 3G,I,J). The protein reduction was also observed in a dose-dependent manner (Figures 3H,K,L), which was in line with the downregulation of gene expression of c-fos and NFATc1 observed in our qPCR analysis (Figures 3A,B). All these results further indicated that BNTA inhibited osteoclastogenesis *in vitro*.

### BNTA inhibited ROS generation induced by RANKL *in vitro* and upregulated SOD1/SOD2 expression both *in vitro* and *in vivo*

**FIGURE 4:**
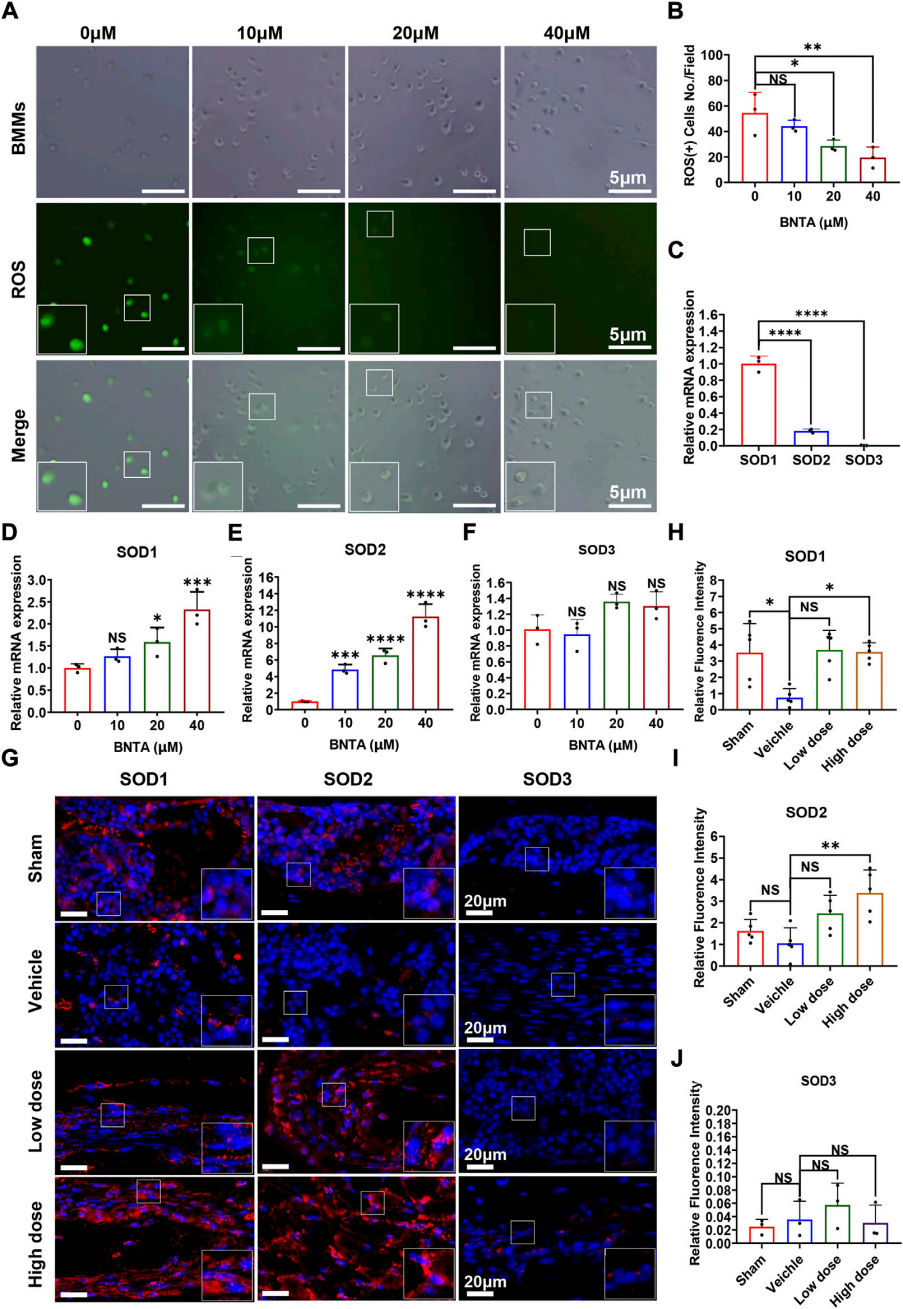
BNTA regulated Redox-related gene expression and inhibited intracellular ROS generation. **(A)** Representative fluorescence images of RANKL-stimulated ROS production in BMMs with or without pre-treatment of indicated dose of BNTA. **(B)** Quantification of the number of ROS positive cells per field (*n* = 3 per group). All data are presented as mean ± SD. *p < 0.05, **p < 0.01. **(C)** qPCR analysis of mRNA expression of SOD1, SOD2, and SOD3 relative to GAPDH in BMMs stimulated by RANKL for 5 days without stimulation of BNTA (*n* = 3 per group). All data are presented as mean ± SD. ****p < 0.0001 compared with control group. **(D–F)** qPCR analysis of redox-related genes expression of SOD1, SOD2, and SOD3 relative to GAPDH in BMMs stimulated by RANKL for 5 days with or without an indicated dose of BNTA (*n* = 3 per group). All data are presented as mean ± SD. *p < 0.05, ***p < 0.001, ****p < 0.0001 compared with control group. **(G)** Representative immunofluorescence staining images of decalcified bone sections for protein expression of SOD1, SOD2, and SOD3. **(H–J)** Quantitative analysis of protein expression of SOD1, SOD2, and SOD3 *in vivo*. *p < 0.05, **p < 0.01 compared with control group.

To understand the potential mechanism of BNTA-dependent of osteoclastogenesis inhibition, we evaluated the effect of BNTA on RANKL-induced intracellular ROS generation and SODs expression. Intracellular ROS was detected as a fluorescent signal derived from the oxidative-sensitive fluorescent probe DCF using fluorescent microscopy. The number of ROS-positive cells was significantly reduced in a dose-dependent manner by BNTA treatment (Figures 4A,B). Additionally, known as ROS scavengers, SODs were found to be involved in the inhibition of ROS-stimulated osteoclastogenesis in this study. The results of qPCR exhibited that SOD1 and SOD2 were enhanced dose-dependently in BMMs induced by RANKL with the treatment of BNTA (Figures 4D,E). But SOD3, another member of the superoxide dismutase family usually highly expressed in specific cells or tissues, did not show noticeable expression variation in BMMs regardless induced by RANKL or treated by BNTA (Figures 4C,F). Furthermore, we investigated the generation of SODs in the LPS-induced inflammatory osteolytic model of the mice calvarias. The immunofluorescence assays were applied to analyze the expression levels of oxidative-related proteins, including SOD1, SOD2, and SOD3. It was noted that BNTA observably enhanced the generation of SOD1 and SOD2 in LPS-induced bone tissue (Figures 4G–I, Supplementary Figure S1A,B). The expression level of SOD3 in bone tissue was very low even induced by LPS (Figures 4G,J, Supplementary Figure S1C). These findings indicate that BNTA is able to upregulate the expression of SOD1 and SOD2 and enhance the scavenging ability of ROS.

### BNTA inhibited osteoclastogenesis via suppressing the MAPK pathway

**FIGURE 5:**
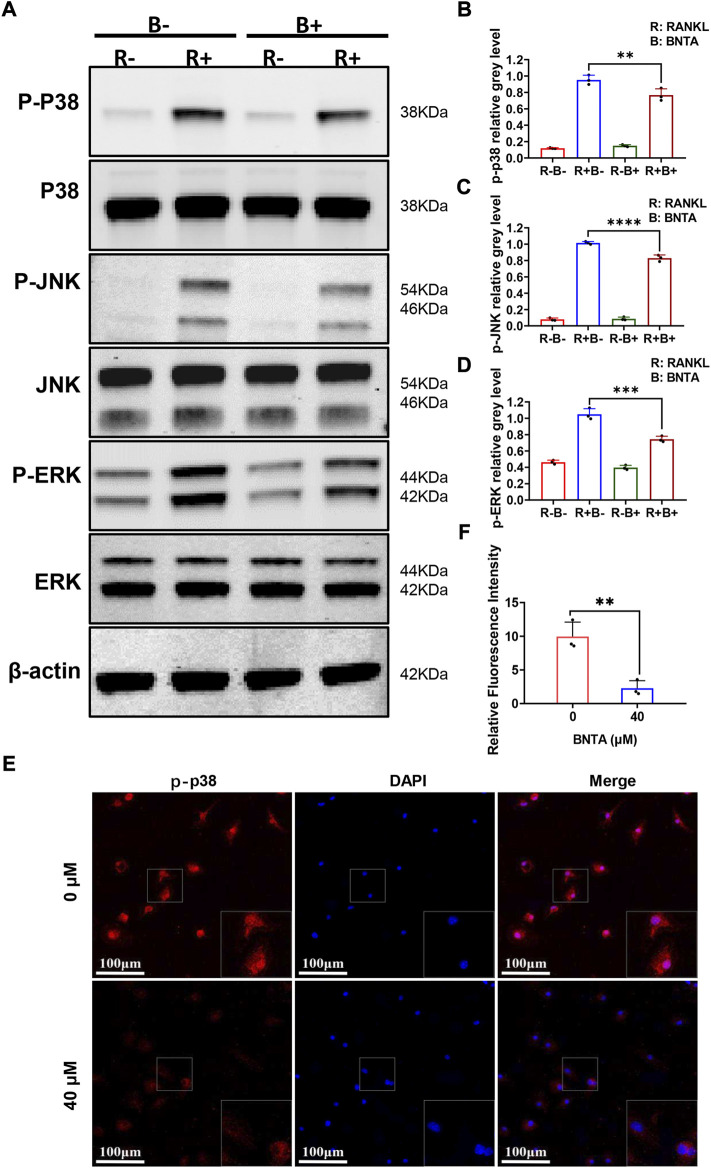
BNTA inhibited osteoclastogenesis via the MAPK signaling pathway. **(A)** Representative Western Blot images of BNTA on the MAPKs pathway, including p38, ERK, and JNK. **(B–D)** Quantification of the ratio of band intensity of p-p38/p38, p-JNK/JNK, p-ERK/ERK (*n* = 3 per group). All data are presented as mean ± SD. **p < 0.01, ***p < 0.001, ****p < 0.0001 compared with control group. **(E)** Representative immunofluorescence images of the effect of BNTA on the phosphorylation and nucleus translocation of p-p38 with stimulation of RANKL. **(F)** Quantification of the ratio of fluorescence intensity (*n* = 3 per group). All data are presented as mean ± SD. **p < 0.01compared with control group.

To further explore the underlying molecular mechanisms of the impact of BNTA on osteoclastogenesis, we investigated the RANKL-stimulated signaling pathways by western blot assay. BMMs were pretreated with 40 μM BNTA for 2 h and then stimulated with RANKL for another 10 min. We observed that phosphorylation of p38, ERK, JNK was upregulated as a result of stimulation of RANKL. However, the phosphorylation could be suppressed obviously by BNTA (Figures 5A–D). Additionally, the immunofluorescence staining revealed that BNTA could significantly inhibit the translocation of phosphorylated p38 from the cytosol to the nucleus (Figures 5E,F). Collectively, these data indicated that BNTA inhibited RANKL-induced osteoclast formation *via* attenuating the activation of the MAPK signaling pathway and subsequent nucleus translocation of p38.

### BNTA attenuated LPS-induced osteolysis and bone loss via modulating osteoclast activity *in vivo*

**FIGURE 6:**
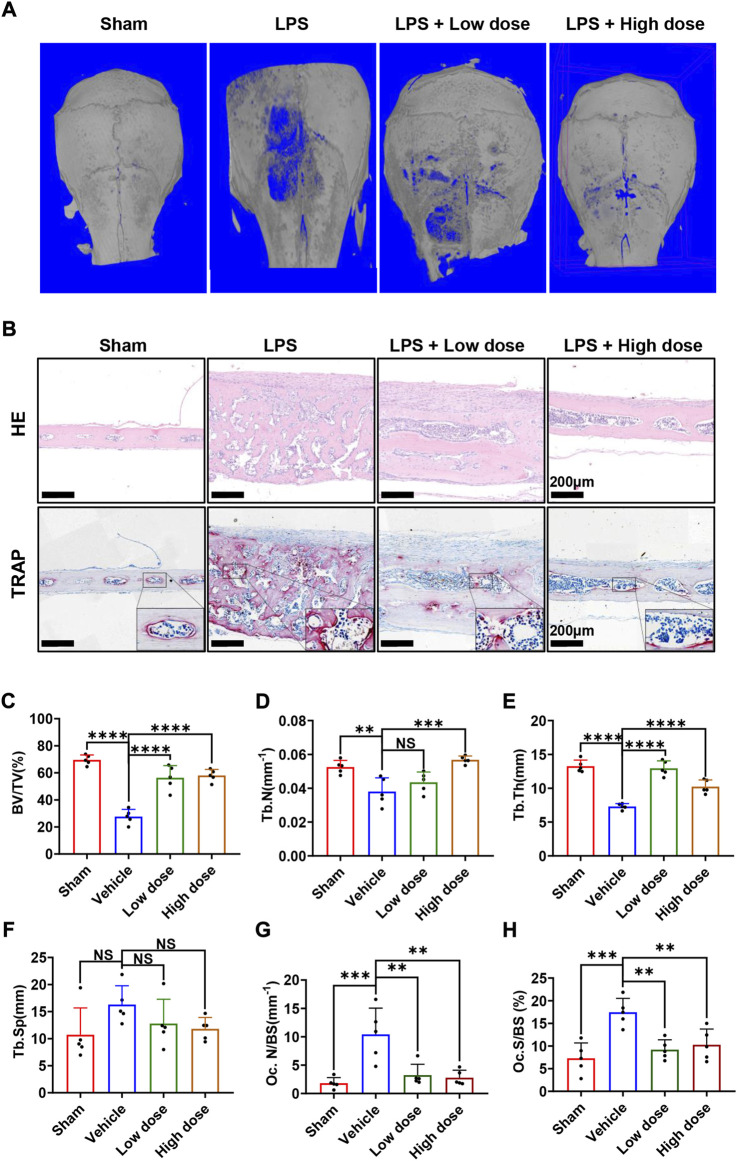
BNTA attenuated LPS-induced bone loss in murine calvaria. **(A)** Micro-CT and 3-dimensional reconstruction of murine calvaria from sham group (PBS), vehicle group (LPS 5 mg/kg body weight), low dose group (LPS 5 mg/kg and BNTA 0.15 mg/kg) and high dose group (LPS 5 mg/kg and BNTA 1.5 mg/kg). **(B)** Representative images of decalcified bone sections stained with H&E and TRAP from each group. **(C–H)** Quantitative analysis of BV/TV, Tb.N, Tb.Th, Tb. sp, Oc. N/BS and Oc. S/BS in tissue sections. (*n* = 5). All data are presented as mean ± SD. **p < 0.01, ***p < 0.001 ****p < 0.0001. TRAP, tartrate resistant acid phosphatase; HE, hematoxylin and eosin; BV/TV, bone volume per tissue volume; Tb.N, trabecular number; Tb.Th, trabecular thickness; Tb. sp, trabecular space; Oc. N/BS, osteoclast number/bone surface; Oc. S/BS, osteoclast surface/bone surface.

Subsequently, the mouse calvarial model of LPS-induced inflammatory osteolysis was used to further evaluate the potential therapeutic application of BNTA toward osteolytic bone loss. The 3D μCT reconstructive images revealed severe osteolysis and widespread bone erosion of varying sizes on the surface of the calvaries in the LPS control group. In contrast, treatment with high-dose BNTA significantly reduced LPS-induced bone destruction in mice (Figure 6A). Quantitative morphometric analysis suggested that BV/TV, Tb.N, Tb.Th in the LPS group were presented to be significantly decreased compared with those in the sham group, while BV/TV, Tb.N and Tb.Th in the high dose treatment group were remarkably ameliorated relative to those in the LPS group (Figures 6C–F). Moreover, histologic and histomorphometry analyses of calvaries further verified the protective effect of BNTA against LPS-induce bone loss. The TRAP-staining assay exhibited that the number of TRAP-positive osteoclasts in the LPS group increased relative to the control group. The formation of osteoclasts and their recruitment to the bone surface was suppressed obviously with the treatment of BNTA (Figures 6B,G,H). Besides, H&E staining showed no significant damage, and toxicity was caused to the normal anatomical structure of main organs including the heart, liver, spleen, lung, and kidney during the treatment with BNTA (Supplementary Figure S3). These data illustrate that BNTA is a potentially effective and safe agent that can prevent bone osteolysis induced by LPS.

### BNTA also regulated the production of proinflammatory cytokines *in vitro* and *in vivo*

**FIGURE 7:**
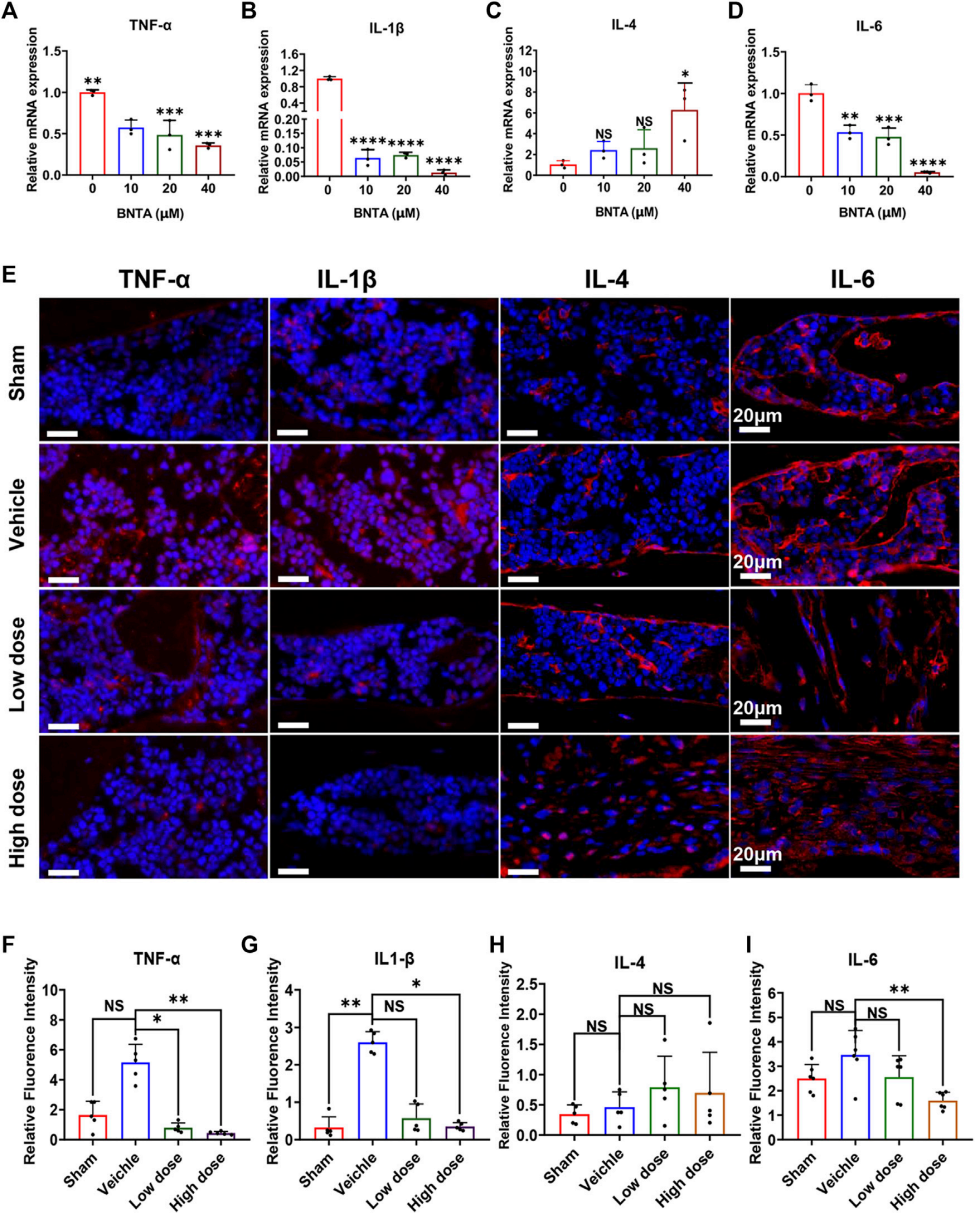
BNTA regulated inflammation-related gene expression. **(A–D)** qPCR analysis of inflammation-related genes expression of TNF-α, IL1-β, IL-4, and IL-6 relative to GAPDH in BMMs stimulated by RANKL for 5 days with or without an indicated dose of BNTA (*n* = 3 per group). All data are presented as mean ± SD. *p < 0.05, **p < 0.01, ***p < 0.001, ****p < 0.0001 compared with control group. **(E)** Representative immunofluorescence staining images of decalcified bone sections for protein expression of TNF-α, IL1-β, IL-4, and IL-6. **(F–I)** Quantitative analysis of protein expression of TNF-α, IL1-β, IL-4, and IL-6 *in vivo* (*n* = 5 per group). All data are presented as mean ± SD. *p < 0.05, **p < 0.01 compared with control group.

Moreover, we inspected the impact of BNTA on inflammatory cytokines in BMMs induced by RANKL. The results found that BNTA markedly suppressed the expression of proinflammatory cytokines, including TNF-α, IL-1β, and IL-6 (Figures 7A,B,D), while increasing the expression of inflammatory inhibitory factors, such as IL-4 (Figure 7C). In addition, these inflammatory cytokines were investigated using immunofluorescence assays as well *in vivo*. The results showed that BNTA significantly inhibited the production of proinflammatory cytokines, including TNF-α, IL-1β, and IL-6, while no influence on the expression of IL-4 (Figures 7E–I, Supplementary Figure S2). These findings indicate that besides promoting the generation of SOD1 and SOD2 and enhancing the scavenging ability of ROS, BNTA is also able to regulate the levels of the inflammatory factors *in vitro* induced by RANKL or *in vivo* stimulated by LPS.

## Discussion

Human bone remodeling is a continuous process throughout life, which is tightly and delicately regulated based on a balance between bone formation by osteoblasts and bone resorption by osteoclasts. Overactivated osteoclastogenesis and excessive bone resorption during the bone remodeling process occur in a variety of inflammatory osteolytic bone diseases, such as osteoarthritis, inflammatory aseptic prosthetic loosening, periprosthetic infection, and osteomyelitis (Roux and Richette, 2012; Gallo et al., 2013; Loi et al., 2016). Current clinically available therapeutic agents for inflammatory osteolysis like estrogen, bisphosphonates, and parathyroid hormone are effective, but still have some limitations and side effects including increased cancer risks, aseptic jaw osteonecrosis, and atypical femur fracture (Clemons and Goss, 2001; Woo et al., 2006; Shane et al., 2014; Nikitovic et al., 2016; Lobo, 2017; Al-Khan et al., 2021). Therefore, the pursuit of novel and effective drug candidates that can safely treat osteolytic bone disorders is always required.

As an artificially synthesized small molecular chemical compound, BNTA identified antioxidative and anti-inflammatory activities in the treatment of OA by enhancing SOD3 expression (Shi et al., 2019). As a result, we consider it might be an effective candidate to treat inflammatory osteolytic bone diseases. In this study, the cytotoxicity assay found that BNTA exhibited no influence on the viability and proliferation of BMM cells, even if the concentration was up to 40 μM. Treated with 40 μM BNTA, the number and area of TRAP-positive osteoclasts were decreased and the resorption area was noticeably reduced, which indicated that BNTA significantly inhibited osteoclastogenesis and osteoclastic resorption. Furthermore, we focused on the expression of osteoclast-related genes. As is suggested, NFATc1 is a key target gene in the phage of osteoclastogenesis stimulated by RANKL (Negishi-Koga and Takayanagi, 2009; Zhao et al., 2010). C-fos, a critical component of the activator protein-1 (AP-1), cooperates with NFATc1 to promote osteoclastic differentiation (Takayanagi et al., 2002; Wagner and Eferl, 2005). This study found that BNTA significantly reduced the expression of c-fos and NFATc1, which further led to the downregulation of associated osteoclast-specific gene expressions including CTSK, D2, MMP9, and ACP5. These findings indicated that BNTA was involved in NFATc1-mediated mechanisms that inhibited osteoclastogenesis and osteoclast resorption.

Next, this study then explored the mechanisms for the anti-oxidative and anti-osteoclastic effects of BNTA. SODs, including SOD1, SOD2, and SOD3, are peroxide catalysts that can catalyze ROS into hydrogen peroxide and then into water, avoiding producing nitrogen oxides, and other toxic substances (Zelko et al., 2002; Shi et al., 2019). Interestingly, we found that BNTA treatment significantly augmented the expression both of SOD1 and SOD2 in BMMs, which attenuated the production of ROS and subsequently inhibited osteoclastic formation. *In vivo* study results also showed upregulation of SOD1 and SOD2 expression with the treatment of BNTA also inhibited osteolytic bone loss. Thus, SOD1 and SOD2 are possibly more important in protecting bone tissue against ROS-mediated osteolytic bone resorption. Additionally, we further explored the underlying signaling pathway. As a physiologic second messenger, ROS can oxidize cysteine residues of proteins, including protein tyrosine phosphatases, and make them inactive. Therefore, ROS may activate MAPKs, which contain threonine and tyrosine residues in their conserved TXY motifs (Lee et al., 2005; Chen K. et al., 2019). The MAPK pathway, including three major family members (p38, JNKs, and ERKs), serves an important role in osteoclast differentiation activated by RANKL (Boyle et al., 2003; Chen K. et al., 2019; He et al., 2021). As signal mediators in osteoclast metabolism, p38, JNKs, and ERKs exhibit differences in the duration of activation. P38 predominantly facilitates osteoclast differentiation and function, whereas JNK and ERK primarily mediate osteoclastic apoptosis and promote osteoclast precursor proliferation, respectively, which indicates that p38 signaling is more tightly connected to the control of osteoclastogenesis and osteoclastic resorption than ERK and JNK signaling (Lee et al., 2018). P38-MAPK signaling is activated by MAPK kinases via phosphorylation of tyrosine and threonine, and subsequently promotes the expression of NFATc1 and osteoclast-related genes, whereas inactivated by MAPK phosphatases (MKPs) through dephosphorylation (Lee et al., 2018). Our research showed that BNTA significantly attenuated the activation of the MAPK signaling pathway and subsequent nucleus translocation of p38. So, BNTA-SOD1/2-ROS-MAPK signaling pathway axis was demonstrated to be involved in regulating osteoclastogenesis.

Furthermore, this study found that BNTA regulated the levels of many inflammatory cytokines during osteoclastogenesis *in vitro* and *in vivo*. Known as a highly conserved important constituent almost of all Gram-negative bacteria, Lipopolysaccharide (LPS) can promote the release of inflammatory factors such as TNF-a and IL-1b, increase the secretion of RANKL, and enhance osteoclast formation and osteolysis (Islam et al., 2007; Guo et al., 2014). Thus, LPS is extensively used in the investigation of inflammatory osteolysis (Islam et al., 2007; Yang et al., 2019; Yang et al., 2021). In our study, LPS was injected on the surfaces of mice’s calvarias to create inflammatory bone erosion models. We inspected that several osteoclasts generated on the inflammatory bone surface, accompanying large amounts of inflammatory cytokines produced and released by multiple immune cells, which was in line with the previous study (Kumar and Roger, 2019). The evidence is clear that some proinflammatory cytokines, like TNF and IL-1, regulate the activation of calcium signaling and NFATc1, which subsequently activate genes related to osteoclastogenesis and osteoclast function (Kim et al., 2013; Terkawi et al., 2022). It is obvious that inflammatory stimulation induces bone resorption and leads to bone loss. As a signaling molecule and a mediator of inflammation, ROS acts a central role in the progression of many inflammatory disorders (Mittal et al., 2014). The nucleotide-binding oligomerization domain (NOD)-like receptor containing pyrin domain 3 (NLRP3) is an inflammasome which was found playing an important role in inflammatory response (Sho and Xu, 2019). Many researchers have confirmed that once it is assembled and activated, the NLRP3 will convert some inflammatory factors precursors such as pro-IL-1β and pro-IL-18 into mature cytokines, leading to inflammatory response in bodies (Pellegrini et al., 2017; Sho and Xu, 2019). ROS has already been detected involving in activation of NLRP3 inflammasome through regulating Caspase-11 ((Lupfer et al., 2014), (Martinon et al., 2002)). In this study we found BNTA significantly promoted the generation of SOD1/SOD2 and then reduced ROS levels in LPS-induced inflammatory wounds. These results suggest that the BNTA-SOD1/2-ROS-inflammatory axis is involved in down-regulating inflammatory activity and attenuating inflammatory osteolysis.

**FIGURE 8:**
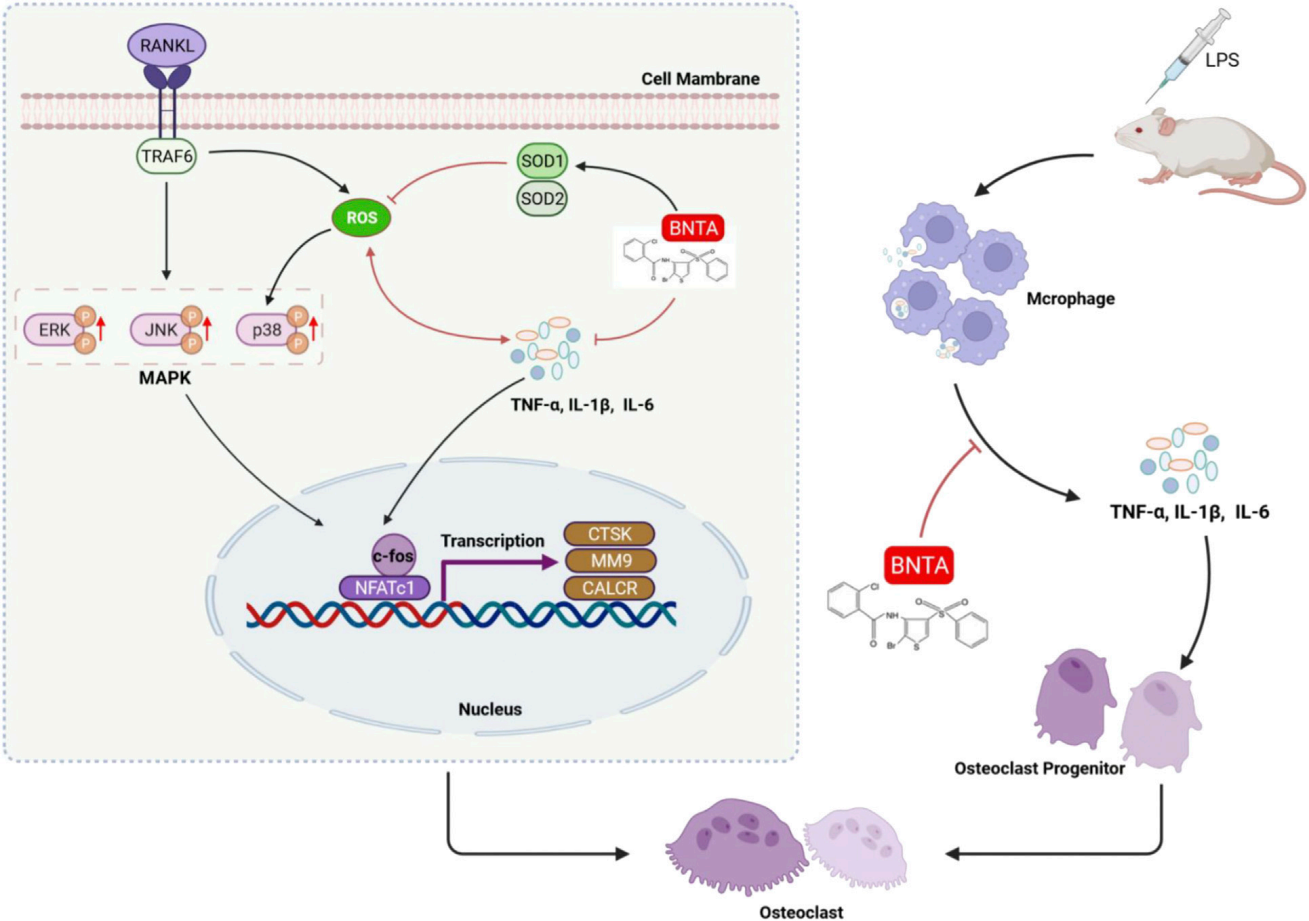
A proposed scheme of BNTA for the inhibitory influence of inflammatory osteoclastogenesis and osteolytic bone resorption.

Our study demonstrated that BNTA exerts an effective inhibitory effect on osteoclastogenesis and osteoclastic resorption through SOD1/2-ROS- MAPK signaling pathway axis and SODs-ROS-inflammation axis (Figure 8). However, there are still two limitations. First, although we detected and elucidated the potential mechanism between SOD1/2 and osteoclastogenesis and osteoclastic resorption and inflammation, the details of the interaction between BNTA and SOD1/2 upregulation still need to be further investigated in our following study. Second, although we confirmed the anti-osteolytic and anti-inflammatory effects of BNTA *in vitro* partially, and further confirmed these effects in mouse models, we still lack a rat model to further elucidate the therapeutic effects of BNTA and we will do further studies in the next experiments.

In summary, this study provides a less cytotoxicity and side effects small molecule compound BNTA, and we confirmed that it acts as an anti-osteoclastic and anti-inflammatory agent in inflammatory osteolytic bone diseases. Moreover, given the advantage of simple chemical structure of BNTA, high yields may be more available if used clinically in the future, which is another benefit. Therefore, the BNTA may be considered as a potential and therapeutic candidate for the prevention and treatment of inflammatory osteoclast-related bone diseases.

## Data Availability

The original contributions presented in the study are included in the article/Supplementary Material, further inquiries can be directed to the corresponding authors.

## Supplementary material

SUPPLEMENTARY FIGURE S1 Representative images of immunofluorescence for SOD1, SOD2, and SOD3 in murine calvaria.**(A,B)** BNTA upregulated protein expression of SOD1 and SOD2. **(C)** The protein expression of SOD3 in murine calvaria was at a low level. Red fluorescence represents marked protein expression. Blue fluorescence represents the cell nucleus.

SUPPLEMENTARY FIGURE S2 Representative images of immunofluorescence for inflammatory factors in murine calvaria. **(A-C)** The protein expression of pro-inflammatory factors of TNF-α, IL1-β, and IL-6 was stimulated by LPS while downregulated by BNTA. **(D)** The protein expression of inflammatory inhibitory factors of IL-4 did not present significant change with the stimulation of LPS or treatment of BNTA. Red fluorescence represents marked protein expression. Blue fluorescence represents the cell nucleus.

SUPPLEMENTARY FIGURE S3 Toxicity analysis in major organs of mice. H&E staining of heart, liver, spleen, lung, and kidney after 10-days treatment of each group.
