# Supplementary figures and images for: BNTA alleviates inflammatory osteolysis by the SOD mediated anti-oxidation and anti-inflammation effect on inhibiting osteoclastogenesis
Source: Front Pharmacol. 2022 Sep 29;13:939929. doi: 10.3389/fphar.2022.939929 (PMC9559729; doi:10.3389/fphar.2022.939929)

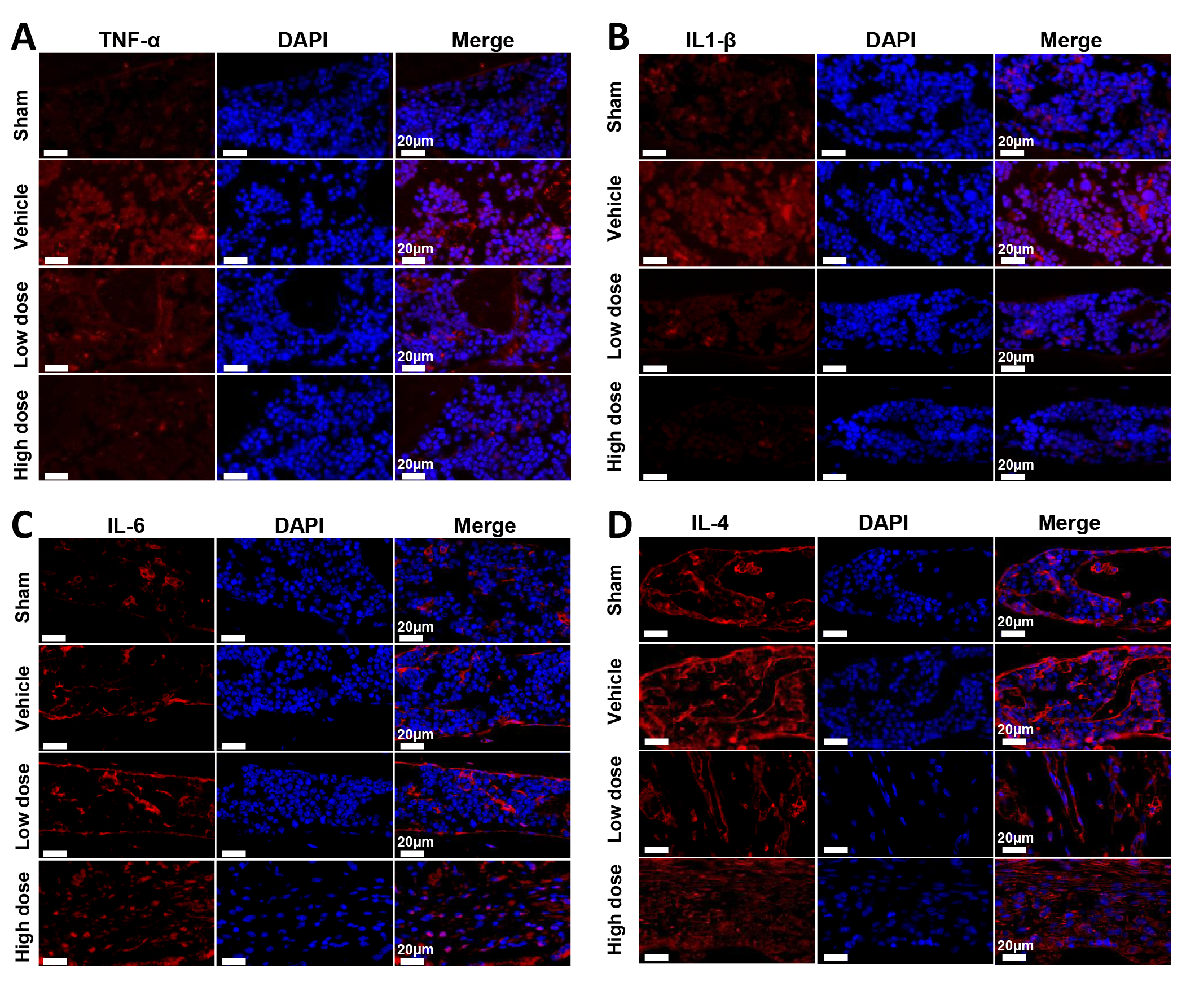

Supplement: Supplementary file 1 [file DataSheet1.zip › Figure S2.tif]

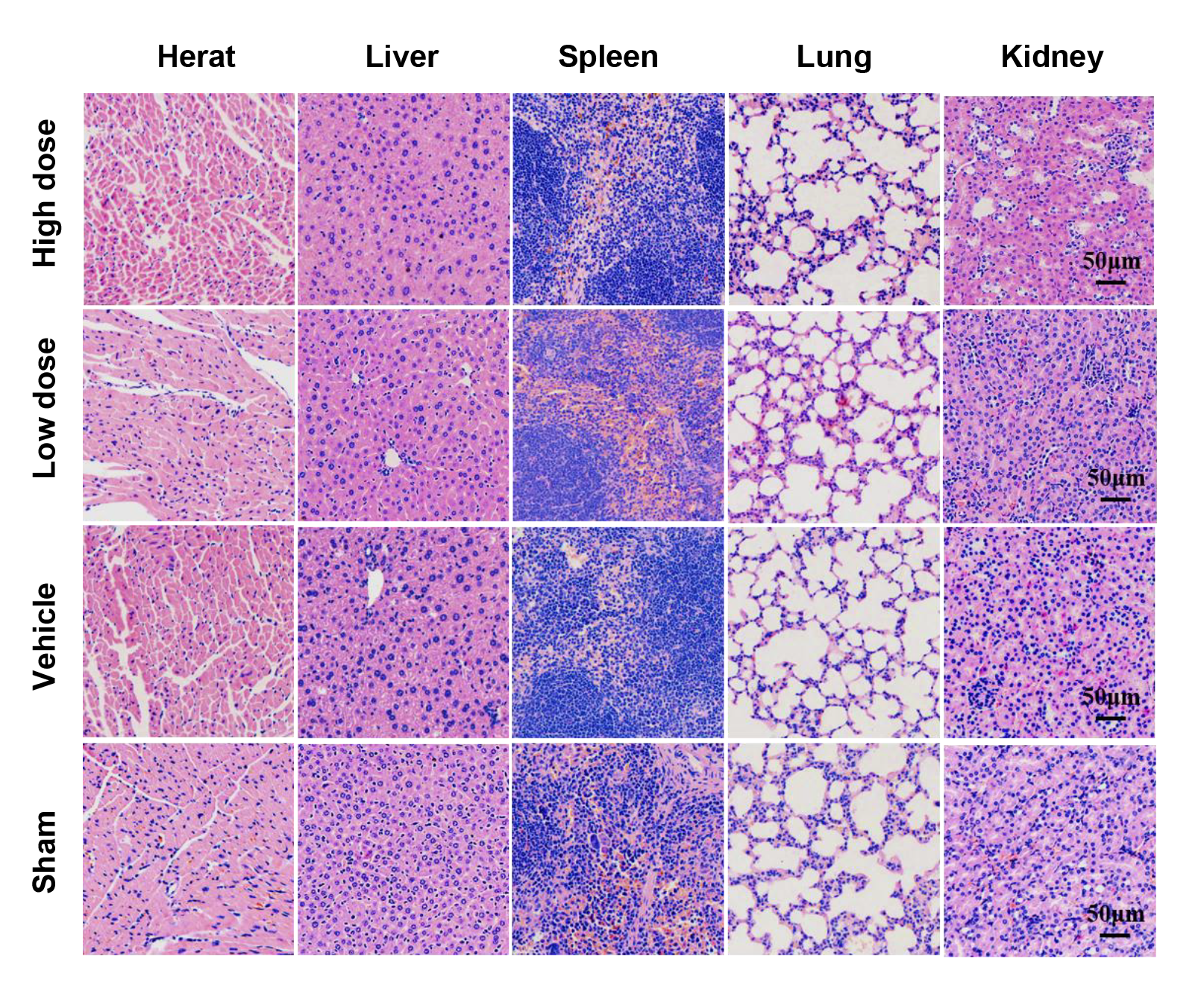

Supplement: Supplementary file 1 [file DataSheet1.zip › Figure S3.tif]

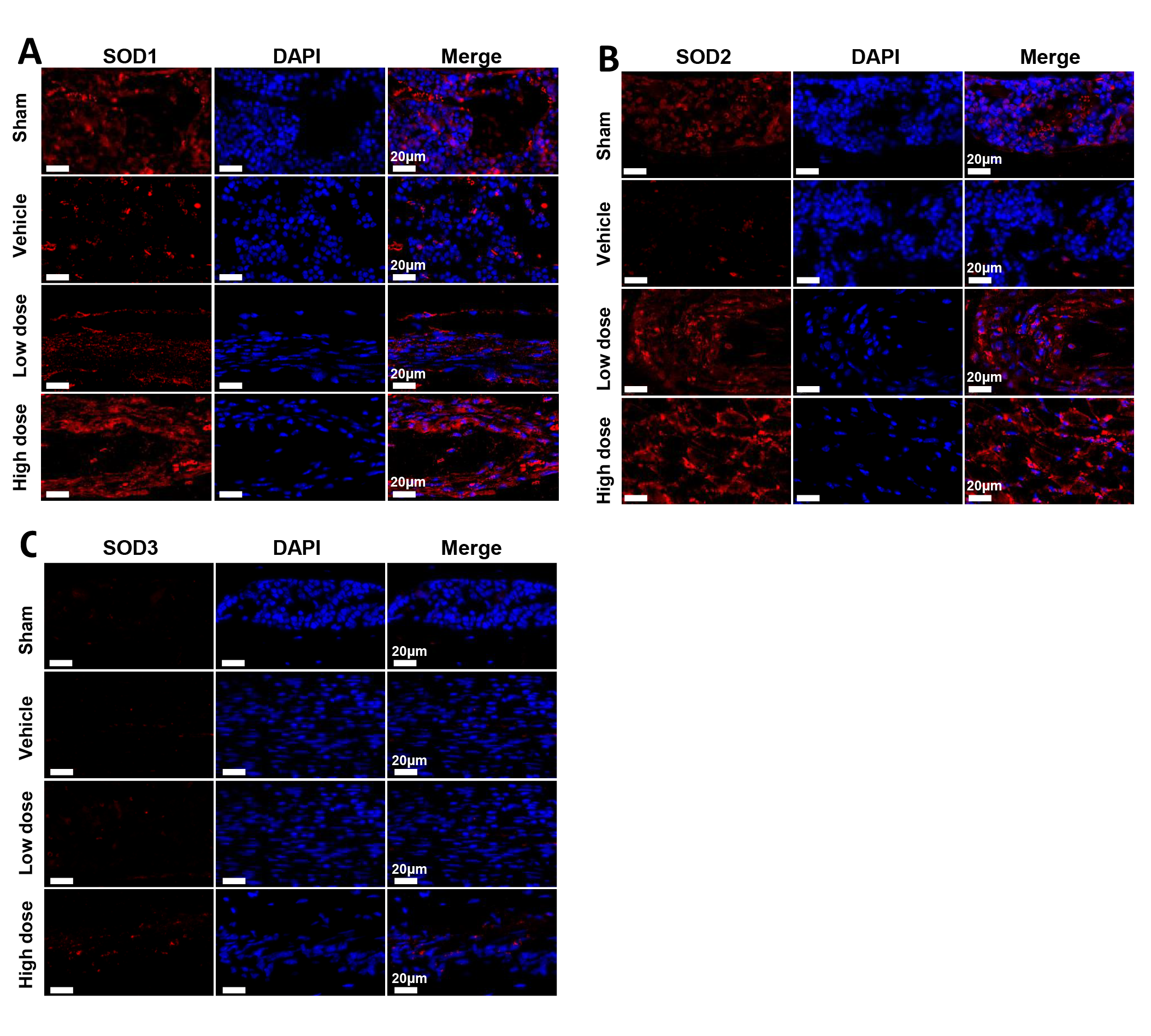

Supplement: Supplementary file 1 [file DataSheet1.zip › Figure S1.tif]
